# Nomogram for predicting the risk and prognosis of lung metastasis of four subtypes of breast cancer: A population-based study from SEER

**DOI:** 10.1016/j.cpt.2024.08.001

**Published:** 2024-08-03

**Authors:** Yuanfang Xin, Guoxin Zhang, Qiuxia Dong, Yaobang Liu, Xingfa Huo, Yumei Guan, Yonghui Zheng, Qianqian Fang, Dengfeng Ren, Fuxing Zhao, Zitao Li, Xinlan Liu, Jiuda Zhao

**Affiliations:** a Breast Disease Diagnosis and Treatment Center of Affiliated Hospital of Qinghai University & Affiliated Cancer Hospital of Qinghai University, Xining, Qinghai 810000, China; b The Second Ward of Oncology, Qinghai Red Cross Hospital, Xining, Qinghai 810000, China; c Department of Medical Oncology, General Hospital of Ningxia Medical University, Yinchuan, Ningxia 750000, China; d Department of Surgical Oncology, General Hospital of Ningxia Medical University, Yinchuan, Ningxia 750000, China; e Precision Medicine Center of Oncology, The Affiliated Hospital of Qingdao University, Qingdao, Shandong 266035, China

**Keywords:** Breast cancer, Lung metastasis, Nomogram, Prognosis, Surveillance, Survival prediction model

## Abstract

**Background:**

Breast cancer (BC) is the most diagnosed cancer worldwide, and patients' survival decreases with metastasis. We conducted a retrospective study using data derived from the Surveillance, Epidemiology, and End Results (SEER) database and clinicopathological data to construct a clinical predictive model to predict the risk and prognosis of lung metastasis (LM) in patients with different subtypes of BC and validate its performance.

**Methods:**

A total of 1650 patients from the SEER database between 2011 and 2015 were enrolled in this study. Cox regression analysis was performed to identify prognostic factors for breast cancer lung metastasis (BCLM). A nomogram was constructed using the independent prognostic factors. The concordance index (C-index), area under the curve (AUC) value, calibration curve, and decision curve analysis (DCA) were used to test the prediction accuracy of the nomogram. External validation (*n* = 112) was performed using clinical data from the Affiliated Hospital of Qinghai University and the General Hospital of Ningxia Medical University.

**Results:**

Multivariate Cox regression analyses suggested that age, grade, surgery, chemotherapy, subtype, and liver, bone, and brain metastases were independent prognostic factors for overall survival (OS). Kaplan–Meier survival analysis showed that the median survival times of patients with human epidermal growth factor receptor 2 (HER2)-positive, luminal A, luminal B, and triple-negative BC were 25 (95% confidence interval [CI], 20–37), 27 (95% CI, 23–29), 35 (95% CI, 30–44), and 12 (95% CI, 11–14), respectively. The C-indexes of the nomogram for predicting OS of the SEER training, SEER validation, and clinical validation cohorts were 0.7, 0.6, and 0.6, respectively, and the calculated AUCs at 3 years were 0.765, 0.794, and 0.799, respectively. The calibration curve indicates that the nomogram possessed a high level of accuracy.

**Conclusions:**

Our nomogram demonstrates significant predictive value, indicating that molecular subtypes, brain metastasis, and liver metastasis are closely associated with the prognosis of patients with LM. This information can guide clinical practice.

## Highlights

• Factors affecting lung metastasis and survival outcomes in breast cancer were explored using Surveillance, Epidemiology, and End Results (SEER) and clinical data.
• Molecular subtypes, brain metastasis, and liver metastasis are closely associated with lung metastasis of breast cancer.
• Through internal and external validation, our model demonstrates good predictive performance.

## Graphical abstract

**Figure:**
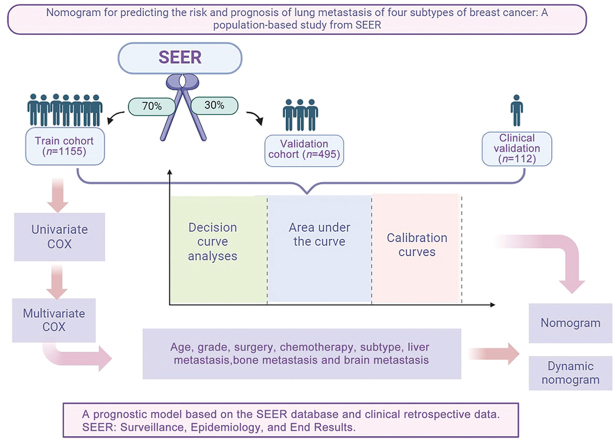

## Introduction

Female breast cancer (BC) is the most diagnosed cancer worldwide.^1^ Primary early BC is considered curable; however, the development of distant metastasis is concerning, owing to the risk of death.^2^^,^^3^ Approximately 4–8% of patients with BC have distant metastasis at the time of diagnosis, and 30% of patients with BC will develop distant metastatic disease.^4^, ^5^, ^6^ The 5-year survival rate for patients with primary BC is 99%, but it drops significantly to 26% for patients with distant metastasis.^7^ The most common sites of BC metastasis are the bone, lung, and liver, whereas brain metastases are considered the final stage of metastasis with a poor prognosis.^8^^,^^9^ Effective control and blocking of tumor metastasis may help reduce the mortality of patients with BC.

The metastasis pattern and prognosis of various subtypes of BC differ and are closely related to estrogen receptor (ER), progesterone receptor (PR), and human epidermal growth factor receptor 2 (HER2) status. The bone is a favorable metastatic site for hormone receptor (HR)-positive tumors, but less suitable for triple-negative breast cancer (TNBC),^5^ which is likely to metastasize to the lungs.^10^ Most patients with BC have no obvious clinical manifestations at the time of lung metastases (LMs), and thus the incidence of LM at first diagnosis of BC cannot be accurately estimated. To prevent cancer metastasis and improve the survival rate of patients with metastatic BC, the clinical and pathological features of metastatic BC should be elucidated. A concurrent understanding of the differences in metastatic patterns may aid in the diagnosis and treatment of BC metastasis.^8^

The prognosis of metastatic BC is related to many factors, including age, sex, race, histological grade, pathological type, molecular type, tumor stage, chemotherapy, radiotherapy, and surgery.^8^ Accurate assessment of prognosis in patients with LM is becoming increasingly important for selecting the most effective treatment. Therefore, we conducted a retrospective study based on data from the Surveillance, Epidemiology, and End Results (SEER) database and clinicopathological data of patients with BC with LM from the Affiliated Hospital of Qinghai University and the General Hospital of Ningxia Medical University. We constructed a nomogram of LM probability for patients with different BC subtypes to predict the risk of metastasis and the prognosis of the patients. The influence of other metastatic sites and the number of metastatic sites on the prognosis of patients were also evaluated.

## Methods

### Data collection and study design

**Figure 1:**
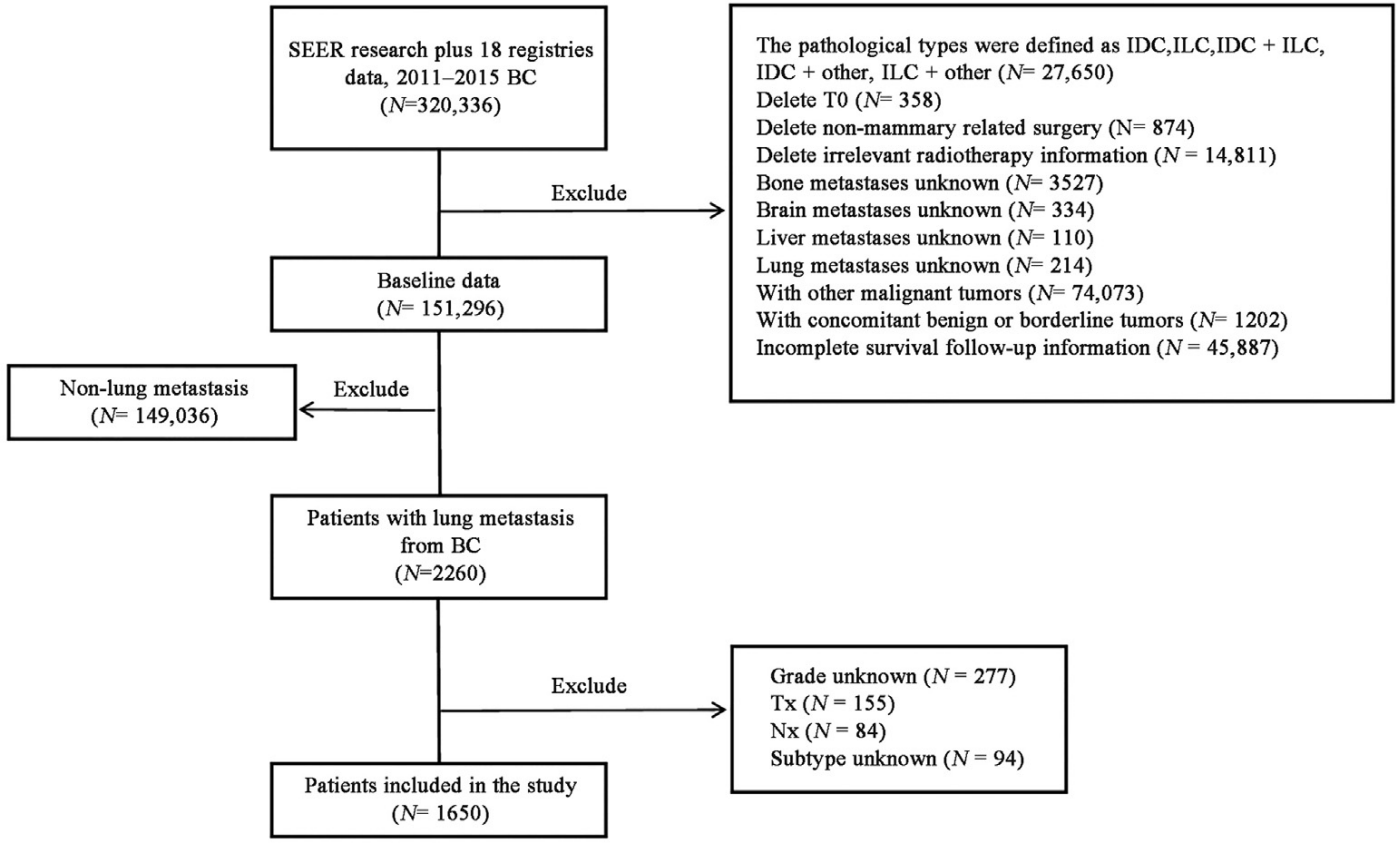
The flowchart of included patients. BC: Breast cancer; IDC: Infiltrating duct carcinoma; ILC: Infiltrating lobular carcinoma; SEER: Surveillance, Epidemiology, and End Results.

We downloaded population-based data from 18 registries in the SEER research database (SEER∗Stat Version 8.4.0). Data on primary invasive BC cases with LM were collected from January 2011 to December 2015; this period was selected because data on molecular subtypes and metastatic sites of BC were not included until 2010. The inclusion criteria were as follows: patients with BC as the first and primary cancer, BC diagnosed between 2011 and 2015, ≥20 years of age, data on molecular subtypes and distant metastatic sites (lung, bone, brain, and liver), and survival data >0 days. The exclusion criteria were patients diagnosed by autopsy or death certificate, follow-up of unknown patients, those with other malignancies, those with occult BC (T0), and those with unspecified T and N stages. The study flowchart is shown in Figure 1.

Before conducting this study, we applied for data access in the SEER's official website and extracted the following data: age at diagnosis (≥20 years), sex (male or female), year of diagnosis (2011–2015), race (American Indian/Alaska Native, Asian or Pacific Islander, Black, White), histopathological type (infiltrating duct carcinoma [IDC], infiltrating lobular carcinoma [ILC], IDC + ILC, IDC + others, ILC + others), histological grading (grade I, grade II, grade III/IV, unknown), derived American Joint Committee on Cancer (AJCC) T stage (T1–T4, Tx), derived AJCC N stage (N0–N3, Nx) (tumor node metastasis classification (TNM) version 6.0), surgery (yes or no), chemotherapy (yes or no), radiotherapy (yes or no), LM (yes or no), liver metastasis (yes or no), brain metastasis (yes or no), bone metastasis (yes or no), breast carcinoma molecular subtypes (HER2-enriched, luminal A, luminal B, triple negative), SEER cause-specific death classification, survival months, and vital status (alive or dead). Informed consent was not required to obtain these data. Overall survival (OS) was defined as the time from initial diagnosis of BC to death (from any cause).

Clinicopathological data were collected from the Affiliated Hospital of Qinghai University and the General Hospital of Ningxia Medical University between January 2014 and December 2017. The data collected were similar to those collected from the SEER database; inclusion and exclusion criteria were also applied similarly. The collection of follow-up data was completed in December 2022.

### Statistical analysis

The chi-squared test was used to analyze the incidence of LM in different BC subgroups and baseline characteristics of all patients. All patients with LM were divided into training and validation sets at a ratio of 7:3. Univariate Cox regression was performed to determine the prognostic factors associated with LM in patients with BC: age, sex, pathological type, grade, molecular subtype, AJCC T, AJCC N, surgery, chemotherapy, radiotherapy, and liver, brain, and bone metastasis. Variables with *P* < 0.05 were included in the multivariate Cox regression analysis to determine the independent prognostic factors in patients with LM. The significant variables (*P* < 0.05) on multivariate Cox regression analysis were screened to construct a nomogram. In addition, we developed a dynamic nomogram to enhance the practicability and accuracy of the nomogram. Based on the webpage, clinicians can select different variables to output survival probabilities and 95% confidence intervals (CI).

The nomogram was used to predict the 1-, 2-, and 3-year OS. The performance of the nomogram was evaluated by calculating the concordance index (C-index), which ranged from 0.5 to 1.0; the higher the C-index, the higher the accuracy of the prediction model. In addition, the receiver operating characteristic (ROC) curve was used to calculate the area under the curve (AUC) to evaluate the performance of the nomogram. The AUC values ranged from 0.5 to 1.0; the closer the AUC value to 1, the more accurate the prediction results of the model. The consistency between the predicted and actual survival rates of patients with LM at 1, 2, and 3 years was evaluated by constructing calibration curves. Decision curve analysis (DCA) was used to more comprehensively assess the performance of prognostic models in clinical decision-making. The Kaplan–Meier survival curve was used to evaluate the survival of patients with LM from different BC subtypes, and the survival rate was tested using the log-rank test. *P* value < 0.05 was considered statistically significant. All statistical analyses were performed using the R software (version 4.1.3, http://www.r-project.org). R software was developed by Ross Ihaka and Robert Gentleman at the University of Auckland, New Zealand.

## Results

### Demographic and clinicopathological characteristics

We downloaded 151,296 cases of BC from the SEER database based on our inclusion and exclusion criteria. After excluding cases with unclear molecular typing, a total of 143,563 patients were analyzed. Baseline data were summarized according to LM of various BC subtypes [Supplementary Table 1]. Among them, the incidence rates of different molecular subtypes in all BC patients are as follows: luminal A, 104,520 cases (72.8%); luminal B, 16,363 cases (11.4%); HER2-enriched, 6887 cases (4.8%); and triple-negative, 15,793 cases (11%). The incidence rates of LM are 1.05%, 2.35%, 3.45%, and 2.35% respectively for luminal A, luminal B, HER2-enriched subtype, and triple-negative subtype. Among them, the probability of LM in patients with brain metastasis was significantly higher in patients with HER2-positive subtype than in those with triple-negative, luminal B, and luminal A subtypes (3.0%, 2.0%, 2.1%, and 1.0%, respectively). A higher incidence of LM was observed in patients with HER2-positive BC without surgery, chemotherapy, or radiotherapy (19.8%, 4.0%, or 4.8%, respectively). Patients with HER2-enriched BC had a higher incidence of lung cancer, regardless of the T and N stages or histological grade.

### Characteristics of the surveillance, epidemiology, and end results training; surveillance, epidemiology, and end results validation; and clinical validation cohorts

A total of 1762 patients with LM were included after excluding cases with missing variables. There were 1155, 495, and 112 cases in the SEER training, SEER validation, and clinical validation cohorts, respectively. The median follow-up duration for all three cohorts was 18 months. The patients were aged between 40 and 79 years, and IDC was the most common pathological type. The proportion of patients with histological grades III/IV was higher in the SEER training and validation cohorts (55.3% vs 62.2%), while grade II was higher in the clinical cohort (56.3%). The SEER training and validation cohorts had a higher proportion of luminal A subtype (51.9% and 46.9%), but a smaller proportion of HER2-enriched subtype (11.1% and 12.1%). Luminal B was more common in the clinical validation cohort (56.3%). Most patients in the clinical cohort underwent surgery and chemotherapy (81.3% vs. 94.6%), whereas in the SEER cohort, most patients did not undergo surgery, and the proportion of patients receiving chemotherapy was also lower compared to the clinical cohort. A significantly lower proportion of patients received radiation therapy in all cohorts. In addition, among the patients with LM of BC, the highest incidence of other metastasis was bone metastases (55.6%), followed by liver metastases (31.3%), whereas brain metastases had the lowest incidence (9.9%). The characteristics of the SEER training, SEER validation, and clinical validation cohorts are shown in Table 1.

**Table 1:** Demographic and clinicopathological characteristics of the SEER training, SEER validation, and clinical validation cohorts with BCLM.

Variables	SEER train cohort, *n* (%) (*N* = 1155)	SEER validation cohort, *n* (%) (*N* = 495)	Clinical validation cohort, *n* (%) (*N* = 112)
Sex			
Female	1136 (98.4)	486 (98.2)	109 (97.3)
Male	19 (1.6)	9 (1.8)	3 (2.7)
Age (years)			
20–39	71 (6.1)	39 (7.9)	11 (9.8)
40–59	445 (38.5)	189 (38.2)	64 (57.1)
60–79	513 (44.4)	212 (42.8)	35 (31.3)
≥80	126 (10.9)	55 (11.1)	2 (1.8)
Histology			
IDC	1073 (92.9)	458 (92.5)	101 (90.2)
ILC	36 (3.1)	16 (3.2)	2 (1.8)
IDC + ILC	33 (2.9)	14 (2.8)	0 (0)
IDC + others	12 (1.0)	7 (1.4)	3 (2.7)
ILC + others	1 (0.1)	0 (0)	0 (0)
Grade			
I	57 (4.9)	21 (4.2)	0 (0)
II	459 (39.7)	166 (33.5)	63 (56.3)
III/IV	639 (55.3)	308 (62.2)	23 (20.5)
Unknown	0 (0)	0 (0)	26 (23.2)
AJCC.T			
T1	93 (8.1)	46 (9.3)	17 (15.2)
T2	341 (29.5)	137 (27.7)	56 (50.0)
T3	208 (18.0)	102 (20.6)	7 (6.3)
T4	513 (44.4)	210 (42.4)	20 (17.9)
Tx	0 (0)	0 (0)	11 (9.8)
AJCC.N			
N0	227 (19.7)	90 (18.2)	22 (19.6)
N1	600 (51.9)	264 (53.3)	35 (31.3)
N2	144 (12.5)	66 (13.3)	18 (16.1)
N3	184 (15.9)	75 (15.2)	23 (20.5)
Nx	0 (0)	0 (0)	13 (11.6)
Subtype			
HER2 enriched	128 (11.1)	60 (12.1)	16 (14.3)
Luminal A	599 (51.9)	232 (46.9)	15 (13.4)
Luminal B	212 (18.4)	113 (22.8)	63 (56.3)
Triple negative	216 (18.7)	90 (18.2)	14 (12.5)
Unknown	0 (0)	0 (0)	4 (3.6)
Surgery			
No	838 (72.6)	344 (69.5)	21 (18.8)
Yes	317 (27.4)	151 (30.5)	91 (81.3)
Chemotherapy			
No/unknown	449 (38.9)	180 (36.4)	6 (5.4)
Yes	706 (61.1)	315 (63.6)	106 (94.6)
Radiation			
None/unknown	801 (69.4)	337 (68.1)	70 (62.5)
Yes	354 (30.6)	158 (31.9)	42 (37.5)
Liver.M			
No	794 (68.7)	328 (66.3)	60 (53.6)
Yes	361 (31.3)	167 (33.7)	52 (46.4)
Bone.M			
No	513 (44.4)	234 (47.3)	46 (41.1)
Yes	642 (55.6)	261 (52.7)	66 (58.9)
Brain.M			
No	1041 (90.1)	449 (90.7)	90 (80.4)
Yes	114 (9.9)	46 (9.3)	22 (19.6)

AJCC.N: American Joint Committee on Cancer N stage; AJCC.T: American Joint Committee on Cancer T stage; BCLM: Breast cancer lung metastasis; Bone.M: Bone metastasis; Brain.M: Brain metastasis; HER2: Human epidermal growth factor receptor 2; IDC: Infiltrating duct carcinoma; ILC: Infiltrating lobular carcinoma; Liver.M: Liver metastasis; SEER: Surveillance, Epidemiology, and End Results.

### Univariate and multivariate Cox regression analyses

Univariate Cox regression analysis showed that age, tumor grade, AJCC T stage, surgery, chemotherapy, subtype, liver metastasis, bone metastasis, and brain metastasis were associated with the prognosis of patients with LM of BC (*P* < 0.001). On multivariate Cox regression analysis, histological grades III/IV had a significantly higher risk of LM than grade I (hazard ratio [HR], 1.93; 95% confidence interval [CI], 1.35–2.76; *P* < 0.001). Patients who received surgery (HR, 0.72; 95% CI, 0.61–0.85; *P* < 0.001) and chemotherapy (HR, 0.59; 95% CI, 0.51–0.69; *P* < 0.001) had significantly improved OS. Compared with HER2-enriched, luminal B subtype (HR, 0.67; 95% CI, 0.51–0.88; *P* = 0.004) was associated with a better prognosis, while triple-negative subtype (HR, 2.27; 95% CI, 1.75–2.93; *P* < 0.001) had a poor prognosis. When LM was combined with liver metastasis (HR, 1.75; 95% CI, 1.51–2.03; *P* < 0.001), bone metastasis (HR, 1.33; 95% CI, 1.14–1.55; *P* < 0.001), or brain metastasis (HR, 2.53; 95% CI, 2.01–3.19; *P* < 0.001), it was associated with increased mortality, particularly with the presence of brain metastases [Table 2 and Supplementary Figure 1].

**Table 2:** Univariate and multivariate Cox regression analyses results in the SEER training cohort.

Variables	Value*, n* (%)	Univariable	Multivariable	Final
		HR (95% CI, *P*)	HR (95% CI, *P*)	HR (95% CI, *P*)
Age (years)				
20–39	71 (6.1)			
40–59	445 (38.5)	1.25 (0.92–1.70, 0.158)	1.53 (1.12–2.10, 0.008)	1.54 (1.13–2.10, 0.007)
60–79	513 (44.4)	1.51 (1.12–2.05, 0.008)	1.84 (1.35–2.52, <0.001)	1.86 (1.36–2.54, <0.001)
≥80	126 (10.9)	2.46 (1.75–3.46, <0.001)	3.39 (2.36–4.86, <0.001)	3.38 (2.36–4.83, <0.001)
Sex				
Female	1136 (98.4)			
Male	19 (1.6)	0.84 (0.50–1.39, 0.491)	0.81 (0.48–1.37, 0.435)	
Race				
American Indian/Alaska Native	8 (0.7)			
Asian or Pacific Islander	89 (7.7)		1.02 (0.44–2.39, 0.958)	1.04 (0.45–2.42, 0.930)
Black	275 (23.8)		1.45 (0.64–3.29, 0.374)	1.49 (0.66–3.37, 0.334)
Unknown	2 (0.2)		0.00 (0.00–Inf, 0.983)	0.00 (0.00–Inf, 0.983)
White	781 (67.6)		1.11 (0.49–2.51, 0.794)	1.13 (0.50–2.54, 0.763)
Histology				
IDC	1073 (92.9)			
IDC+ILC	33 (2.9)	0.70 (0.46–1.07, 0.099)	0.89 (0.58–1.38, 0.610)	
IDC+others	12 (1.0)	0.73 (0.39–1.36, 0.319)	0.81 (0.43–1.54, 0.529)	
ILC	36 (3.1)	1.20 (0.83–1.72, 0.337)	1.26 (0.87–1.84, 0.225)	
ILC+others	1 (0.1)	3.66 (0.51–26.09, 0.195)	7.28 (1.00–53.06, 0.050)	
Grade				
I	57 (4.9)			
II	459 (39.7)	1.33 (0.95–1.87, 0.095)	1.41 (1.00–2.00, 0.052)	1.37 (0.97–1.93, 0.070)
III/IV	639 (55.3)	1.74 (1.25–2.44, 0.001)	1.93 (1.35–2.76, <0.001)	1.89 (1.34–2.67, <0.001)
AJCC.T				
T1	93 (8.1)			
T2	341 (29.5)	1.27 (0.96–1.68, 0.097)	1.13 (0.85–1.51, 0.382)	
T3	208 (18.0)	1.43 (1.06–1.92, 0.018)	1.18 (0.88–1.60, 0.271)	
T4	513 (44.4)	1.54 (1.18–2.01, 0.002)	1.27 (0.96–1.69, 0.092)	
AJCC.N				
N0	227 (19.7)			
N1	600 (51.9)	0.94 (0.79–1.12, 0.468)	0.94 (0.79–1.14, 0.545)	
N2	144 (12.5)	0.84 (0.66–1.07, 0.152)	0.95 (0.73–1.22, 0.682)	
N3	184 (15.9)	0.94 (0.75–1.17, 0.560)	0.87 (0.68–1.10, 0.239)	
Surgery				
No	838 (72.6)			
Yes	317 (27.4)	0.69 (0.60–0.81, <0.001)	0.72 (0.61–0.85, <0.001)	0.70 (0.60–0.82, <0.001)
Chemotherapy				
No/unknown	449 (38.9)			
Yes	706 (61.1)	0.65 (0.57–0.74, <0.001)	0.59 (0.51–0.69, <0.001)	0.59 (0.50–0.68, <0.001)
Radiation				
None/unknown	801 (69.4)			
Yes	354 (30.6)	1.11 (0.97–1.28, 0.138)	0.93 (0.79–1.09, 0.379)	
Bone.M				
No	513 (44.4)			
Yes	642 (55.6)	1.23 (1.07–1.40, 0.003)	1.33 (1.14–1.55, <0.001)	1.30 (1.12–1.51, 0.001)
Liver.M				
No	794 (68.7)			
Yes	361 (31.3)	1.69 (1.47–1.94, <0.001)	1.75 (1.51–2.03, <0.001)	1.79 (1.55–2.07, <0.001)
Brain.M				
No	1041 (90.1)			
Yes	114 (9.9)	2.60 (2.12–3.18, <0.001)	2.53 (2.01–3.19, <0.001)	2.46 (1.99–3.04, <0.001)
Subtype				
HER2 enriched	128 (11.1)			
Luminal A	599 (51.9)	1.00 (0.80–1.25, 0.978)	0.91 (0.71–1.17, 0.468)	0.92 (0.72–1.18, 0.500)
Luminal B	212 (18.4)	0.70 (0.54–0.91, 0.009)	0.67 (0.51–0.88, 0.004)	0.67 (0.51–0.87, 0.003)
Triple negative	216 (18.7)	1.99 (1.55–2.55, <0.001)	2.27 (1.75–2.93, <0.001)	2.22 (1.72–2.86, <0.001)

*N* = 1155, Events = 893, Likelihood-ratio test = 409.69 on 29 df (*P* < 0.001). AJCC.N: American Joint Committee on Cancer N stage; AJCC.T: American Joint Committee on Cancer T stage; Bone.M: Bone metastasis; Brain.M: Brain metastasis; CI: Confidence interval; df: Degree of freedom; HER2: Human epidermal growth factor receptor 2; HR: Hazard ratio, IDC: Infiltrating duct carcinoma; ILC: Infiltrating lobular carcinoma; Inf: Infinity; Liver.M: Liver metastasis.

### Construction and validation of the nomogram

**Figure 2:**
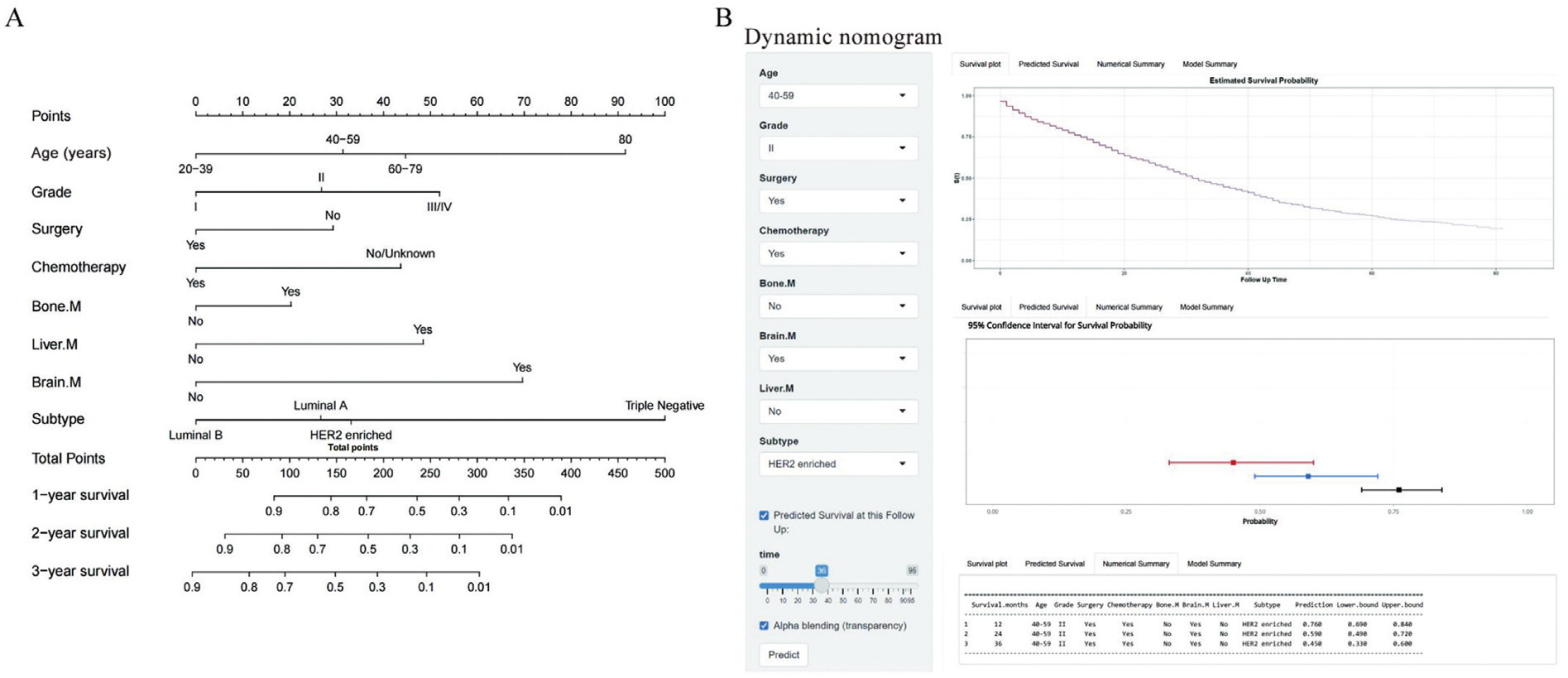
Nomogram predicts 1-, 2-, and 3-year overall survival (OS) in patients with lung metastases. (A) Survival nomogram. (B) Dynamic nomogram. Bone.M: Bone metastasis; Brain.M: Brain metastasis; HER2: HER2: Human epidermal growth factor receptor 2; Liver.M: Liver metastasis; OS: Overall survival.

Multivariate Cox regression analysis suggested that age, tumor grade, surgery, chemotherapy, subtype, liver metastasis, bone metastasis, and brain metastasis were independent prognostic factors for OS; therefore, a nomogram of LM probability was constructed to predict the 1-, 2-, and 3-year OS [Figure 2]. The 1-year survival rates of patients with HER2-positive, luminal A, luminal B, and triple-negative BC were 64.7%, 69.0%, 75.9%, and 48.4%, respectively; the 3-year survival rates were 42.0%, 38.6%, 48.6%, and 11.7 %, respectively; and the 5-year survival rates were 26.5%, 18.1%, 35.9%, and 6.3%, respectively.

The nomogram suggested that patients with lung and brain metastases of BC had the worst prognosis, followed by those with liver and bone metastases. Patients with LMs of TNBC had worse survival outcomes than those with LMs of other BC subtypes. Patients with histological grade III tumors had a poor prognosis. Prognostic factors in patients with BC with LM were older age, lower tumor differentiation, hormone receptor and HER2 expression, and metastasis to other sites.

**Figure 3:**
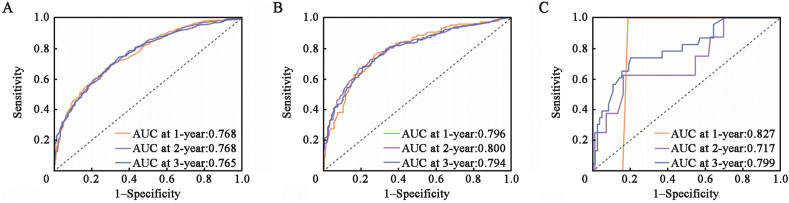
The 1-, 2-, and 3-year ROC curve validation nomogram. (A) 1-, 2-, 3-year survival in the SEER training cohort (B) 1-, 2-, and 3-year survival in the SEER validation cohort (C) 1-, 2-, and 3-year survival in the clinical validation cohort. AUC: Area under the curve; ROC: Receiver operating characteristic; SEER: Surveillance, Epidemiology, and End Results.

**Figure 4:**
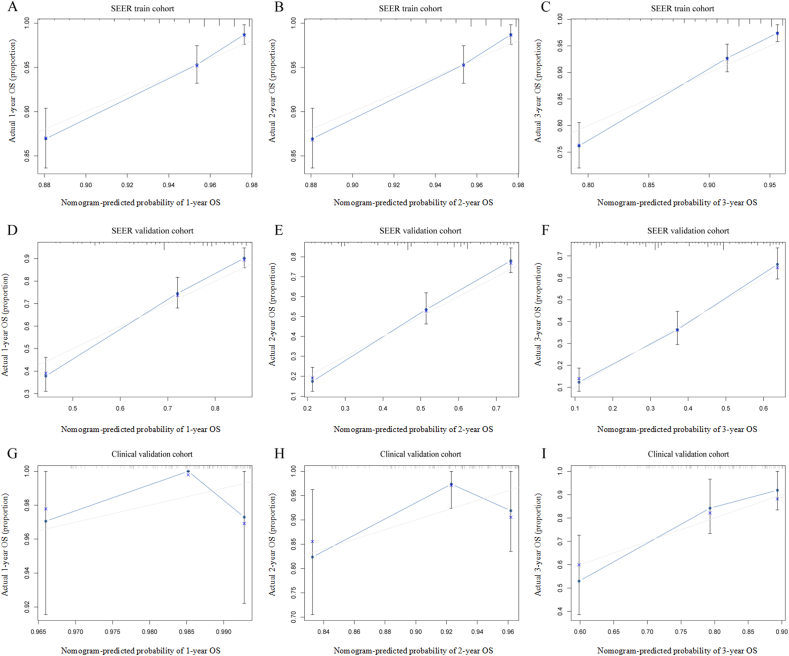
Calibration curves of nomogram evaluated 1-, 2-, and 3-year predicted survival outcomes and actual survival outcomes in the training and validation cohorts. (A–C) 1-, 2-, and 3-year survival in the SEER training cohort. (D–F) 1-, 2-, and 3-year survival in the SEER validation cohort. (G–I) 1-, 2-, and 3-year survival in the clinical validation cohort. OS: Overall survival; SEER: Surveillance, Epidemiology, and End Results.

**Figure 5:**
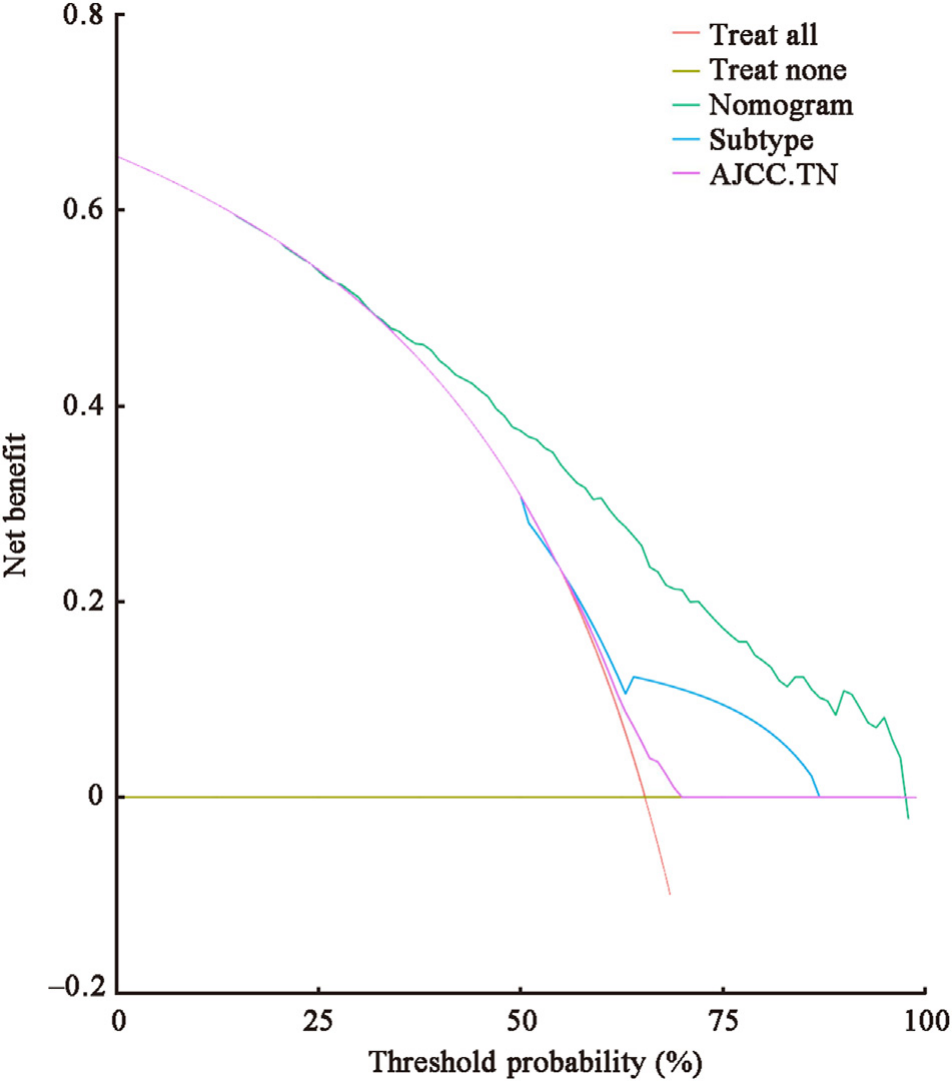
Decision curve analysis (DCA) of the nomogram, subtype, and AJCC.TNM staging system for 3-year overall survival. AJCC.TN: American Joint Committee on Cancer TN stage; AJCC.TNM: American Joint Committee on Cancer TNM stages; DCA: Decision curve analysis.

**Figure 6:**
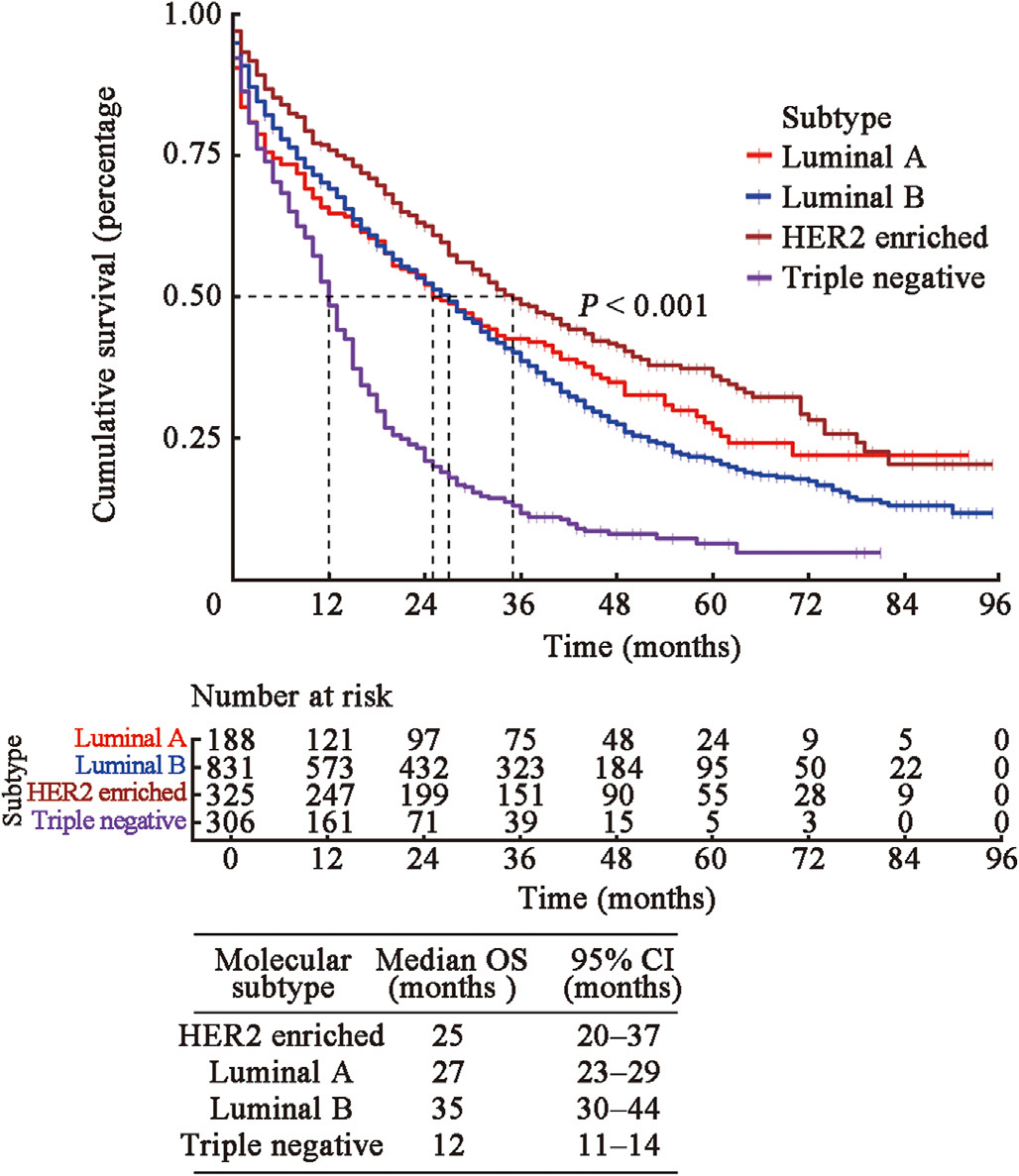
The Kaplan–Meier survival curve was used to compare the overall survival of patients with different subtypes of lung metastasis. HER2: Human epidermal growth factor receptor 2; OS: Overall survival.

The C-indexes of the nomogram for predicting OS in the SEER training, SEER validation, and clinical validation cohorts were 0.7, 0.6, and 0.6, respectively, indicating moderate prediction accuracy of the nomogram for OS. In addition, 1-, 2-, and 3-year AUC values of the SEER training, SEER validation, and clinical validation cohorts were 0.768, 0.796, and 0.827; 0.768, 0.800, and 0.717; and 0.765, 0.794, and 0.799, respectively, indicating a good predictive effect of the model [Figure 3]. Calibration curves showed a high degree of agreement between the 1-, 2-, and 3-year predicted and actual survival outcomes in the SEER training cohort, but a slightly poor agreement in the clinical validation cohort, possibly because of the small sample size in the clinical validation cohort [Figure 4]. We applied DCA to assess the clinical utility of the nomogram in predicting LM of BC. The DCA results demonstrated that compared to AJCC-TNM staging and molecular subtypes, our nomogram had better net benefit across different threshold probabilities [Figure 5]. Kaplan–Meier survival analysis showed that the median survival times of patients with HER2-positive, luminal A, luminal B, and triple-negative BC were 25 months (95% CI, 20–37), 27 months (95% CI, 23–29), 35 months (95% CI, 30–44), and 12 months (95% CI, 11–14), respectively [Figure 6].

## Discussion

This study analyzed the risk factors and prognosis of LM in patients with different subtypes of BC, based on the SEER and clinical data. Some previous studies have explored the prognostic model of LM based on the SEER data; however, they mainly used gene-level data,^11^^,^^12^ predicted survival outcomes of LM in patients with BC,^13^^,^^14^ or constructed a prognosis model of LM for a specific molecular subtype.^15^ To the best of our knowledge, this is the first study to explore the effects of different BC subtypes, stages, pathology, and treatments on LM of BC and survival outcomes based on SEER and multicenter clinical retrospective data. Based on univariate and multivariate Cox regression analysis, we found that age, grade, surgery, chemotherapy, molecular subtype, and liver, bone, and brain metastases are significantly associated with the occurrence of lung metastasis in breast cancer. Simultaneously, we constructed a nomogram and validated it using internal and external data. This model can be used to assess the patients with BC at risk of LMs, thereby enhancing their surveillance.

A total of 143,563 patients with BC between 2011 and 2015 were included in this study. The highest incidence of LMs was observed for HER2-positive BC (3.5%, 238/6887) and the lowest incidence for luminal A BC (1.1%, 1102/104,520). In patients with liver metastases, the probabilities of LMs for HER2-positive, luminal A, luminal B, and triple-negative BCs were 2.7%, 1.0%, 2.1%, and 2.3%, respectively, whereas those for patients with bone metastases were 3.1%, 1.1%, 2.5%, and 2.5%, respectively, and those for patients with brain metastases were 3.5%, 1.0%, 2.2%, and 2.4%, respectively. Based on these results, molecular subtypes are considered to be associated with the incidence and survival of patients with LMs of BC. The increased incidence of LM in patients with liver, bone, or brain metastases of different BC subtypes can be attributed to several factors. First, different subtypes of BC have unique clinical characteristics and recurrence and metastasis patterns, displaying specific mechanisms of metastasis.^16^ Second, the LM of BC may be mediated by specific genes.^17^ Different tumor subtypes carry specific gene mutations and molecular features that may promote their growth and spread to specific organs. Additionally, the tumor microenvironment plays a crucial role in tumor cell metastasis, and tumor cells exhibit organ-specific metastatic tendencies. The interactions within the tumor microenvironments of the liver, bones, and brain may promote metastasis, while the rich vascular network in the lungs makes it easier for tumor cells to colonize.

Our study also showed that patients with HER2-positive BC had the highest incidence of liver (4.5%, 310/6887) and brain metastases (1.0%, 67/6887). The highest incidence of bone metastases was observed in patients with HER2-positive and luminal B subtypes (4.5%, 313/6887 *vs*. 4.9%, 799/16,363). The results are consistent with previous findings.^9^^,^^18^^,^^19^ However, a previous study reported the highest incidence of liver metastases with luminal A.^20^

Age, histological grade, surgery, chemotherapy, molecular subtype, and liver, bone, and brain metastases were independent prognostic factors in patients with LMs of BC in our study. The prognosis was worse for patients with brain metastases, followed by liver and bone metastases. These findings are consistent with previous reports that bone metastases are associated with better survival and brain metastases with poorer survival.^9^^,^^21^ Patients with TNBC have a poor prognosis, which is associated with high aggressiveness, heterogeneity, and recurrence rate.^22^^,^^23^ Patients with luminal B subtype have the best prognosis, which may be related to various treatment methods, such as anti-HER2 therapies, chemotherapy, and endocrine treatment.

Surgery for primary BC is beneficial for patient prognosis. The surgical treatment of the primary tumor can improve the survival of patients with metastatic BC by reducing tumor burden.^24^, ^25^, ^26^ Our study results showed that chemotherapy has a certain benefit on the prognosis. Chemotherapy can reduce the risk of BC recurrence and metastasis, particularly in patients with TNBC and HER2-positive subtypes.^27^ However, some studies report a survival benefit in only ER-negative patients.^28^^,^^29^ Tumor size did not significantly contribute to the prognosis of patients with LM of BC. Patients with poor histological differentiation also had a worse prognosis. The histological grade has been reported to significantly correlate with the LM of BC (*P* = 0.013).^30^ Patients aged 20–39 years have a good prognosis. A study exploring the relationship between age and distant metastasis of BC showed that the risk of LM increases with age.^31^

The lung is a common metastatic site in patients with BC, and the survival rate of these patients is substantially reduced. The Kaplan–Meier survival analysis showed significant survival differences among various molecular subtypes of BC. The median survival times of patients with HER2-positive, luminal A, luminal B, and triple-negative BCs with LMs were 25, 27, 35, and 12 months, respectively.

The nomogram predicted the 1-, 2-, and 3-year survival probabilities of patients with LMs of BC, and internal and external validation was performed using training and validation cohorts. The AUC value, C-index, calibration curve, and DCA were used to verify the performance of the nomogram. The validation results suggested that our model had a good predictive effect. However, the actual survival curve for the clinical validation cohort was slightly less consistent with the predicted survival curve. These results may be explained by the small sample size of the clinical validation cohort. In addition, the SEER database contains 30% of the US population and is thus ethnically and therapeutically biased against Asia. In summary, our nomogram may help patients monitor metastasis and treatment in clinical practice, but the results should be interpreted with caution.

The study had some limitations. First, the marker of proliferation Ki-67 (Ki67) and HER2 expression values, as well as fluorescence *in situ* hybridization (FISH) results, were missing. As a result, we directly extracted molecular subtype data from the SEER database without subdividing the data according to the hormone receptor and HER2 status, which may bias our results. Second, our study was retrospective, and thus the nomogram requires further validation using prospective data. Third, the SEER database does not provide detailed information on chemotherapy regimens, surgical modality, radiotherapy site, dose, anti-HER2-targeted therapy, endocrine therapy for hormone receptor-positive patients, and other biologics, and thus may be biased in assessing survival benefits. In addition, the SEER database lacked information on metastasis and recurrence during follow-up. As such, patients with partial recurrence or LM during follow-up were not included in this study, which may have biased our results. The SEER database does not record treatment details for patients with LMs; therefore, caution should be exercised in survival assessments.

In conclusion, age, histological grade, surgery, chemotherapy, molecular subtypes, and liver, brain, and bone metastases were independent prognostic factors in patients with LM of BC. We developed a traditional and dynamic nomogram to predict patient survival rates. Emphasis should be placed on the molecular subtype, histological grade, and presence of liver, brain, or bone metastasis while predicting prognosis. In particular, patients with TNBC with brain or liver metastasis should be closely monitored for LMs, thus ensuring early clinical detection and treatment and improving the prognosis and quality of life of the patients. It can serve as a personalized forecasting tool to assist clinicians in accurately predicting patient prognosis and improving the quality of clinical decision-making. Additionally, it is crucial to validate these results in future prospective studies.

## Authors contribution

Yuanfang Xin contributed to writing – original draft; Fuxing Zhao and Xingfa Huo were responsible for writing – reviewing & editing; Yumei Guan and Yonghui Zheng contributed to handle data visualization; Dengfeng Ren and Yaobang Liu conducted the formal analysis; Zitao Li, Qiuxia Dong, Qianqian Fang, and Guoxin Zhang participated in data curation; Jiuda Zhao and Xinlan Liu were responsible for conceptualizing the study, supervision, and validation; Jiuda Zhao also contributed to the editing and responsible for resolving the differences of opinion between the authors. All authors read and approved the final manuscript.

## Ethics statement

The study strictly complied with the requirements of the *Helsinki Declaration* of 1975, as revised in 2000. The clinical data for this study was approved by the Ethics Committees of the Affiliated Hospital of Qinghai University(No. P-SL-2022-071) and the General Hospital of Ningxia Medical University(No. KYLL-2023-0191). Because this was a retrospective study and the clinical validation cohorts data analysis was performed anonymously, this study was exempt from informed consent from patients. Additionally, part of the data was obtained from the publicly accessible SEER database, for which ethical approval and informed consent were not required. The data came from the SEER database which were authorized to legally obtain. Data are authentic and reliable and have not been copied or sourced from other sources.

## Declaration of generative AI and AI-assisted technologies in the writing process

The authors declare that during the preparation of this work, they used artificial intelligence (AI) and AI-assisted technologies solely to refine and streamline the language. After using this tool/service, the authors reviewed and edited the content as needed and take full responsibility for the content of the publication.

## Funding

This work was supported by the Central Government Guiding Local Scientific and Technological Development Funds and Health Commission for Qinghai Province in China (No. 2018-wjzdx-48).

## Conflict of interest

The authors declare that they have no known competing financial interests or personal relationships that could have appeared to influence the work reported in this paper.

## Data Availability

The datasets used in the current study are available from the corresponding author on reasonable request.
