# Supplementary material for: Nomogram for predicting the risk and prognosis of lung metastasis of four subtypes of breast cancer: A population-based study from SEER
Source: Cancer Pathog Ther. 2024 Aug 3;3(2):154–62. doi: 10.1016/j.cpt.2024.08.001 (PMC11963208; doi:10.1016/j.cpt.2024.08.001)
Supplement: Multimedia component 1 [file mmc1.docx]

**Supplementary data**

**Supplementary Figure 1:** The forest plot of multivariate Cox regression analysis was constructed in the SEER training cohort. AIC: Akaike information criterion; AJCC.T: American Joint Committee on Cancer T stage; HER2: Human epidermal growth factor receptor 2; SEER: Surveillance, Epidemiology, and End Results.


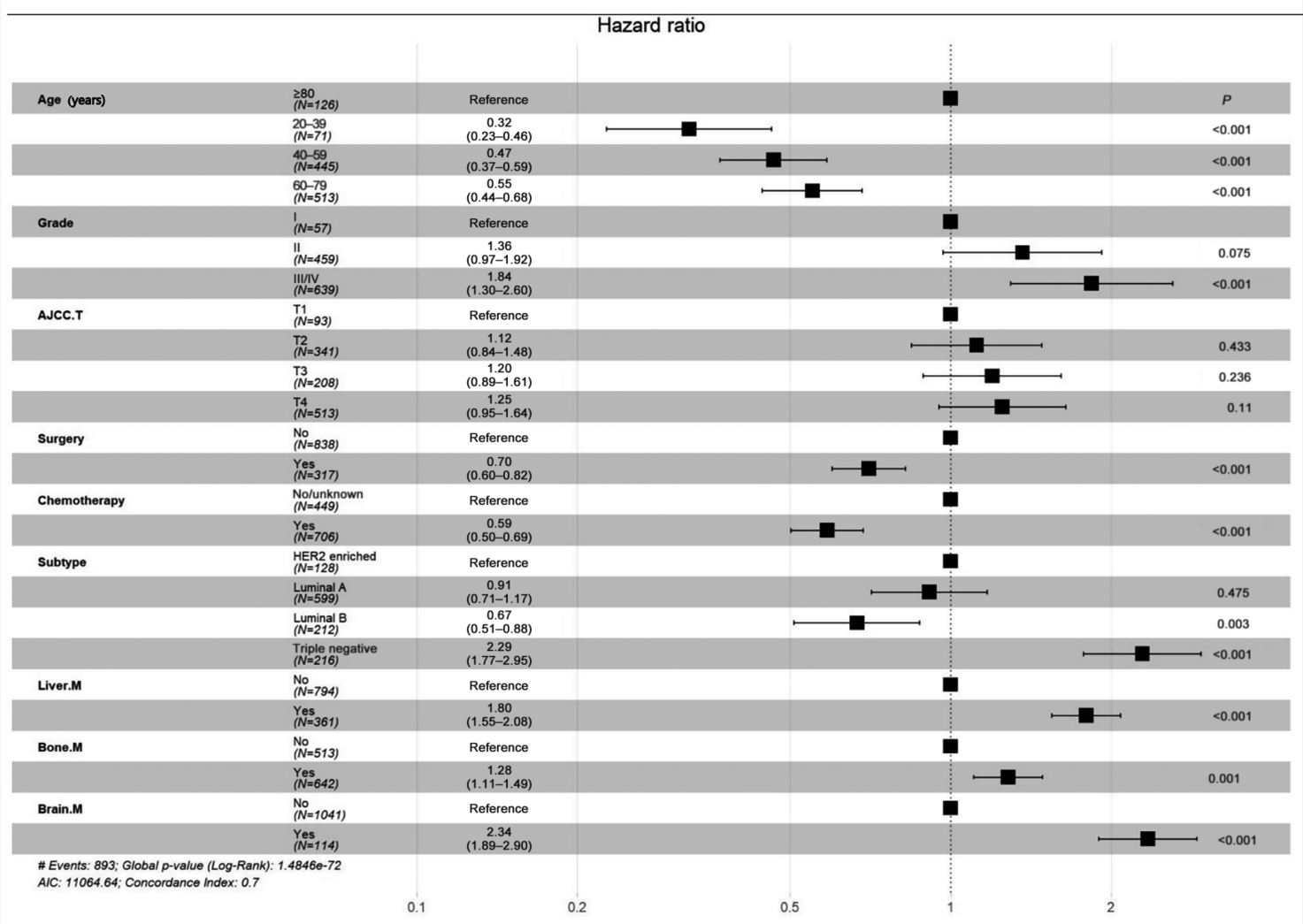


**Supplementary Table 1: Demographic and clinicopathological characteristics of all breast cancers according to molecular subtype.**

| **HER2 enriched** | | | | **Luminal A** | | **Luminal B** | | | **Triple negative** | | |  |
| --- | --- | --- | --- | --- | --- | --- | --- | --- | --- | --- | --- | --- |
| **Variables** | **NN-LM, *n* (%) (*N*=6649)** | **LM, *n* (%)**  **(*N*=238)** | ***P*-value** | **N-LM, *n* (%)**  **(*N*=103,418)** | **LM, *n* (%) (*N*=1102)** | ***P*-value** | **N-LM, *n* (%)**  **(*N*=15,978)** | **LM, *n* (%)**  **(*N*=385)** | ***P*-value** | **N-LM, *n* (%)**  **(*N*=15,421)** | **LM, *n* (%)**  **(*N*=372)** | ***P*-value** |

| **Sex** |  |  |  |  |  |  |  |  |  |  |  |  |
| --- | --- | --- | --- | --- | --- | --- | --- | --- | --- | --- | --- | --- |
| Female | 6640 (99.9) | 238 (100) | <0.001 | 102,615 (99.2) | 1079 (97.9) | <0.001 | 15,880 (99.4) | 380 (98.7) | <0.001 | 15,406 (99.9) | 370 (99.5) | <0.001 |
| Male | 9 (0.1) | 0 (0) |  | 803 (0.8) | 23 (2.1) |  | 98 (0.6) | 5 (1.3) |  | 15 (0.1) | 2 (0.5) |  |
| **Age (years)** |  |  |  |  |  |  |  |  |  |  |  |  |
| ≥80 | 370 (5.6) | 19 (8.0) | <0.001 | 8886 (8.6) | 136 (12.3) | <0.001 | 907 (5.7) | 44 (11.4) | <0.001 | 1044 (6.8) | 43 (11.6) | <0.001 |
| 20–39 | 599 (9.0) | 25 (10.5) |  | 4444 (4.3) | 56 (5.1) |  | 1593 (10.0) | 26 (6.8) |  | 1560 (10.1) | 31 (8.3) |  |
| 40–59 | 3408 (51.3) | 110 (46.2) |  | 43,188 (41.8) | 360 (32.7) |  | 7932 (49.6) | 169 (43.9) |  | 7298 (47.3) | 140 (37.6) |  |
| 60–79 | 2272 (34.2) | 84 (35.3) |  | 46,900 (45.4) | 550 (49.9) |  | 5546 (34.7) | 146 (37.9) |  | 5519 (35.8) | 158 (42.5) |  |
| **Race** |  |  |  |  |  |  |  |  |  |  |  |  |
| American Indian/Alaska Native | 44 (0.7) | 1 (0.4) | <0.001 | 589 (0.6) | 12 (1.1) | <0.001 | 131 (0.8) | 1 (0.3) | <0.001 | 94 (0.6) | 2 (0.5) | <0.001 |
| Asian or Pacific Islander | 832 (12.5) | 23 (9.7) |  | 9158 (8.9) | 79 (7.2) |  | 1574 (9.9) | 40 (10.4) |  | 1100 (7.1) | 28 (7.5) |  |
| Black | 1132 (17.0) | 51 (21.4) |  | 11,320 (10.9) | 235 (21.3) |  | 2267 (14.2) | 78 (20.3) |  | 3713 (24.1) | 107 (28.8) |  |
| Unknown | 40 (0.6) | 1 (0.4) |  | 549 (0.5) | 3 (0.3) |  | 79 (0.5) | 1 (0.3) |  | 69 (0.4) | 0 (0) |  |
| White | 4601 (69.2) | 162 (68.1) |  | 81,802 (79.1) | 773 (70.1) |  | 11,927 (74.6) | 265 (68.8) |  | 10,445 (67.7) | 235 (63.2) |  |
| **Histology** |  |  |  |  |  |  |  |  |  |  |  |  |
| IDC | 6319 (95.0) | 231 (97.1) | <0.001 | 79,721 (77.1) | 970 (88.0) | <0.001 | 14,338 (89.7) | 367 (95.3) | <0.001 | 14,583 (94.6) | 361 (97.0) | <0.001 |
| IDC+ILC | 91 (1.4) | 5 (2.1) |  | 6781 (6.6) | 42 (3.8) |  | 602 (3.8) | 8 (2.1) |  | 184 (1.2) | 3 (0.8) |  |
| IDC+others | 177 (2.7) | 1 (0.4) |  | 3938 (3.8) | 18 (1.6) |  | 415 (2.6) | 4 (1.0) |  | 463 (3.0) | 4 (1.1) |  |
| ILC | 58 (0.9) | 1 (0.4) |  | 12,594 (12.2) | 70 (6.4) |  | 603 (3.8) | 6 (1.6) |  | 171 (1.1) | 4 (1.1) |  |
| ILC+others | 4 (0.1) | 0 (0) |  | 384 (0.4) | 2 (0.2) |  | 20 (0.1) | 0 (0) |  | 20 (0.1) | 0 (0) |  |
| **Grade** |  |  |  |  |  |  |  |  |  |  |  |  |
| I | 90 (1.4) | 0 (0) | <0.001 | 28,412 (27.5) | 74 (6.7) | <0.001 | 892 (5.6) | 11 (2.9) | <0.001 | 218 (1.4) | 4 (1.1) | <0.001 |
| II | 1450 (21.8) | 39 (16.4) |  | 51,036 (49.3) | 516 (46.8) |  | 6351 (39.7) | 124 (32.2) |  | 2498 (16.2) | 40 (10.8) |  |
| III/IV | 4699 (70.7) | 175 (73.5) |  | 20,565 (19.9) | 367 (33.3) |  | 8009 (50.1) | 224 (58.2) |  | 12,173 (78.9) | 294 (79.0) |  |
| Unknown | 410 (6.2) | 24 (10.1) |  | 3405 (3.3) | 145 (13.2) |  | 726 (4.5) | 26 (6.8) |  | 532 (3.4) | 34 (9.1) |  |
| **Stage** |  |  |  |  |  |  |  |  |  |  |  |  |
| I | 2116 (31.8) | 0 (0) | <0.001 | 54,539 (52.7) | 0 (0) | <0.001 | 5862 (36.7) | 0 (0) | <0.001 | 5320 (34.5) | 0 (0) | <0.001 |
| II | 2723 (41.0) | 0 (0) |  | 35,083 (33.9) | 0 (0) |  | 6692 (41.9) | 0 (0) |  | 7003 (45.4) | 0 (0) |  |
| III | 1269 (19.1) | 0 (0) |  | 9694 (9.4) | 0 (0) |  | 2277 (14.3) | 0 (0) |  | 2327 (15.1) | 0 (0) |  |
| IV | 411 (6.2) | 238 (100) |  | 2904 (2.8) | 1102 (100) |  | 857 (5.4) | 385 (100) |  | 539 (3.5) | 372 (100) |  |
| Unknown | 130 (2.0) | 0 (0) |  | 1198 (1.2) | 0 (0) |  | 290 (1.8) | 0 (0) |  | 232 (1.5) | 0 (0) |  |
| **AJCC.T** |  |  |  |  |  |  |  |  |  |  |  |  |
| T1 | 2813 (42.3) | 19 (8.0) | <0.001 | 65,806 (63.6) | 88 (8.0) | <0.001 | 7674 (48.0) | 43 (11.2) | <0.001 | 6614 (42.9) | 19 (5.1) | <0.001 |
| T2 | 2494 (37.5) | 52 (21.8) |  | 28,494 (27.6) | 289 (26.2) |  | 6005 (37.6) | 101 (26.2) |  | 6454 (41.9) | 99 (26.6) |  |
| T3 | 665 (10.0) | 43 (18.1) |  | 5348 (5.2) | 160 (14.5) |  | 1201 (7.5) | 66 (17.1) |  | 1289 (8.4) | 79 (21.2) |  |
| T4 | 522 (7.9) | 103 (43.3) |  | 2458 (2.4) | 450 (40.8) |  | 781 (4.9) | 149 (38.7) |  | 826 (5.4) | 152 (40.9) |  |
| Tx | 155 (2.3) | 21 (8.8) |  | 1312 (1.3) | 115 (10.4) |  | 317 (2.0) | 26 (6.8) |  | 238 (1.5) | 23 (6.2) |  |
| **AJCC.N** |  |  |  |  |  |  |  |  |  |  |  |  |
| N0 | 3600 (54.1) | 45 (18.9) | <0.001 | 72,357 (70.0) | 221 (20.1) | <0.001 | 9344 (58.5) | 64 (16.6) | <0.001 | 9788 (63.5) | 68 (18.3) | <0.001 |
| N1 | 2130 (32.0) | 109 (45.8) |  | 23,123 (22.4) | 547 (49.6) |  | 4834 (30.3) | 202 (52.5) |  | 3855 (25.0) | 177 (47.6) |  |
| N2 | 485 (7.3) | 35 (14.7) |  | 4855 (4.7) | 119 (10.8) |  | 1048 (6.6) | 48 (12.5) |  | 970 (6.3) | 33 (8.9) |  |
| N3 | 386 (5.8) | 34 (14.3) |  | 2508 (2.4) | 129 (11.7) |  | 630 (3.9) | 53 (13.8) |  | 703 (4.6) | 78 (21.0) |  |
| Nx | 48 (0.7) | 15 (6.3) |  | 575 (0.6) | 86 (7.8) |  | 122 (0.8) | 18 (4.7) |  | 105 (0.7) | 16 (4.3) |  |
| **Surgery** |  |  |  |  |  |  |  |  |  |  |  |  |
| No | 670 (10.1) | 166 (69.7) | <0.001 | 5522 (5.3) | 890 (80.8) | <0.001 | 1332 (8.3) | 298 (77.4) | <0.001 | 1086 (7.0) | 242 (65.1) | <0.001 |
| Yes | 5979 (89.9) | 72 (30.3) |  | 97,896 (94.7) | 212 (19.2) |  | 14,646 (91.7) | 87 (22.6) |  | 14,335 (93.0) | 130 (34.9) |  |
| **Chemotherapy** |  |  |  |  |  |  |  |  |  |  |  |  |
| No/unknown | 1406 (21.1) | 59 (24.8) | <0.001 | 71,594 (69.2) | 596 (54.1) | <0.001 | 3959 (24.8) | 111 (28.8) | <0.001 | 3375 (21.9) | 105 (28.2) | <0.001 |
| Yes | 5243 (78.9) | 179 (75.2) |  | 31,824 (30.8) | 506 (45.9) |  | 12,019 (75.2) | 274 (71.2) |  | 12,046 (78.1) | 267 (71.8) |  |
| **Radiation** |  |  |  |  |  |  |  |  |  |  |  |  |
| None/unknown | 3321 (49.9) | 169 (71.0) | <0.001 | 41,369 (40.0) | 771 (70.0) | <0.001 | 7492 (46.9) | 288 (74.8) | <0.001 | 6799 (44.1) | 253 (68.0) | <0.001 |

| **Variables** | **HER2 enriched** | | | **Luminal A** | | | **Luminal B** | | | **Triple negative** | | |
| --- | --- | --- | --- | --- | --- | --- | --- | --- | --- | --- | --- | --- |
|  | **N-LM, *n* (%)**  **(*N*=6649)** | **LM, *n* (%)**  **(*N*=238)** | ***P*-value** | **N-LM, *n* (%)**  **(*N*=103,418)** | **LM, *n* (%) (*N*=1102)** | ***P*-value** | **N-LM, *n* (%)**  **(*N*=15,978)** | **LM, *n* (%)**  **(*N*=385)** | ***P*-value** | **N-LM, *n* (%)**  **(*N*=15,421)** | **LM, *n* (%)**  **(*N*=372)** | ***P*-value** |
| Yes | 3328 (50.1) | 69 (29.0) |  | 62,049 (60.0) | 331 (30.0) |  | 8486 (53.1) | 97 (25.2) |  | 8622 (55.9) | 119 (32.0) |  |
| **Liver.M** |  |  |  |  |  |  |  |  |  |  |  |  |
| No | 6441 (96.9) | 136 (57.1) | <0.001 | 10,2922 (99.5) | 795 (72.1) | <0.001 | 15,669 (98.1) | 236 (61.3) | <0.001 | 15,250 (98.9) | 255 (68.5) | <0.001 |
| Yes | 208 (3.1) | 102 (42.9) |  | 496 (0.5) | 307 (27.9) |  | 309 (1.9) | 149 (38.7) |  | 171 (1.1) | 117 (31.5) |  |
| **Bone.M** |  |  |  |  |  |  |  |  |  |  |  |  |
| No | 6448 (97.0) | 126 (52.9) | <0.001 | 101,090 (97.7) | 355 (32.2) | <0.001 | 15,394 (96.3) | 170 (44.2) | <0.001 | 15,147 (98.2) | 244 (65.6) | <0.001 |
| Yes | 201 (3.0) | 112 (47.1) |  | 2328 (2.3) | 747 (67.8) |  | 584 (3.7) | 215 (55.8) |  | 274 (1.8) | 128 (34.4) |  |
| **Brain.M** |  |  |  |  |  |  |  |  |  |  |  |  |
| No | 6616 (99.5) | 204 (85.7) | <0.001 | 103,281 (99.9) | 1017 (92.3) | <0.001 | 15,922 (99.6) | 347 (90.1) | <0.001 | 15,376 (99.7) | 320 (86.0) | <0.001 |
| Yes | 33 (0.5) | 34 (14.3) |  | 137 (0.1) | 85 (7.7) |  | 56 (0.4) | 38 (9.9) |  | 45 (0.3) | 52 (14.0) |  |

AJCC.N: American Joint Committee on Cancer N stage; AJCC.T: American Joint Committee on Cancer T stage; Bone.M: Bone metastasis; Brain.M: Brain metastasis; HER2: Human epidermal growth factor receptor 2; IDC: Infiltrating duct carcinoma; ILC: Infiltrating lobular carcinoma; Liver.M: Liver metastasis; LM: Lung metastasis; Max: Maximum; Min: Minimum; N-LM: Non-lung metastasis.
